# Sex differences in the contribution of different physiological systems to physical function in older adults

**DOI:** 10.1007/s11357-021-00328-y

**Published:** 2021-02-11

**Authors:** Siana Jones, Martin G. Schultz, Therese Tillin, Chloe Park, Suzanne Williams, Nishi Chaturvedi, Alun D. Hughes

**Affiliations:** 1 MRC Unit for Lifelong Health & Ageing at UCL, Department of Population Science & Experimental Medicine, Institute for Cardiovascular Science, University College London, 5th floor, 1-19 Torrington Place, London, WC1E 7HB UK; 2 Menzies Institute for Medical Research, University of Tasmania, Hobart, Australia

**Keywords:** Physical function, Cardiovascular, Skeletal muscle, Pulmonary

## Abstract

Having the physical function to undertake activities of daily living (ADLs) is essential in order to maintain independence. The aim of this study is to investigate factors associated with physical function in older adults and determine if these associations differ in men versus women. In total, 726 participants (57% men; 73±7 years old) from a population-based cohort, the Southall and Brent Revisited (SABRE) study, completed questionnaires permitting a physical function score (PFS) to be calculated. Detailed phenotyping was performed including cardiovascular (echocardiography and macrovascular and microvascular functions), skeletal muscle (grip strength and oxidative capacity) and lung (pulmonary) function measurements. In a sub-group, maximal aerobic capacity was estimated from a sub-maximal exercise test. In women versus men, the association between grip strength and PFS was nearly 3 times stronger, and the association between microvascular dysfunction and PFS was over 5 times stronger (standardized β-coefficient (95% CI) 0.34 (0.22, 0.45) versus 0.11 (0.01,0.22) and −0.27 (−0.37, −0.17) versus −0.05 (−0.14, 0.04), respectively). In men, the association between cardiorespiratory fitness and PFS was 3 times greater than that in women (standardized β-coefficient (95% CI) 0.33 (0.22, 0.45) versus 0.10 (−0.04, 0.25). Cardiovascular, skeletal muscle and pulmonary factors all contribute to self-reported physical function, but the relative pattern of contribution differs by sex. Grip strength and microvascular function are most strongly associated with physical function in women while cardiorespiratory fitness is most strongly associated with physical function in men. This is relevant to the design of effective interventions that target maintenance of physical function in old age.

**Supplementary Information:**

The online version contains supplementary material available at 10.1007/s11357-021-00328-y.

## Introduction

Physical function (or capability) contributes to an individual’s capacity to undertake activities of daily living (ADLs) and is essential in older adults in order to maintain independence [1, 2]. Off-setting the loss of independence with age reduces disability, the burden of social care and morbidity. In order to develop targeted, or novel, approaches that sustain independent living, a firm understanding of the factors that contribute to poor physical function in older adults is necessary.

Ageing is associated with a decline in cardiorespiratory fitness [3]. Cardiorespiratory fitness at the whole-body level, typically measured as V̇O_2_max, is strongly associated with physical function [4, 5]. It is generally accepted that, in healthy individuals, the primary limiting factor for cardiorespiratory fitness is oxygen delivery by the cardiovascular system [6]. Cardiovascular components can broadly be categorized into cardiac, macrovascular and microvascular functions. Sub-clinical macrovascular measures, intima-media thickness (IMT) and pulse wave velocity (PWV) have been directly linked to physical function (objectively measured by the Short Physical Performance Battery; SBBP) [7].

Other key physiological components contributing to physical function include lung (pulmonary) function and skeletal muscle function. Lung function is widely recognized as a key component of the oxygen transport system. Lung function declines with age leading to impaired ventilation which may negatively affect physical function in older adults [8]. Two common measures of lung function, forced vital capacity (FVC) and forced expired volume in 1 second (FEV1), have recently been linked directly to physical performance [9]. Grip-strength is positively associated with walking speed and composite physical function (a derived outcome comprised of both lower and upper body measures, or self-reported markers, of functional disability) in men and women [10, 11]. Localized oxidative capacity of skeletal muscle, measured directly in muscle (mitochondrial capacity and efficiency), has also been associated with walking speed [4]. While some have suggested that muscle strength could mediate this association [12], few studies have investigated the contributions of oxidative capacity and muscle strength to physical function within one study.

Sex differences in the pattern of cardiovascular aging at the population level [13, 14], and in the clinical manifestations of heart failure [15], are well recognized. Compared with men, women have a higher prevalence of heart failure with preserved ejection fraction (HFPEF). This difference is thought to be underpinned by differential pathophysiology in women versus men; including higher inflammation and greater microvascular and diastolic dysfunction [15]. It is therefore plausible that the relative contribution of cardiovascular factors to overall physical function in older adults differs by sex.

The objectives of this analysis were to (1) describe individual associations between factors within different physiological systems (cardiovascular factors, skeletal muscle factors, and lung function) and self-reported physical function in older adults, (2) compare the relative contribution of these factors to physical function and (3) determine if these associations differ in men versus women.

## Methods

### Participants

Participants were enrolled in a population-based cohort study of older adults resident in West London, UK: the Southall and Brent Revisited (SABRE) study.[16] SABRE originally recruited participants aged 40–69 years in 1988-1991. Data presented in this study were collected at the 25–30-year follow-up visit (2014–2018). Full details are provided in the cohort profile and update [16, 17]. All participants who attended clinic were invited to undertake all measurements apart from the exercise test. Some participants were excluded from undertaking exercise according to co-morbidity contraindications given in the American College of Sports Medicine guidelines [18].

All procedures were in accordance with the principles of the Helsinki declaration, and all participants gave written informed consent, and the study was approved by the National Research Ethics Service (NRES) Committee London—North Fulham.

### Anthropometrics

Height was measured barefoot using a stadiometer (seca 217; seca, Hamburg, Germany). Weight was measured using digital bio-impedance scales (BC-418; Tanita, IL, USA). Body surface area (BSA) was estimated using the Dubois formula. Waist and hip circumferences were measured in cm using a tape measure, and the waist-hip ratio was calculated. Lean mass was measured using a full-body dual energy X-ray absorptiometry (DEXA) scan.

### Questionnaires and physical function

Physical function was assessed from self-reported information provided in questionnaires. A range of questions were included to provide information about 2 aspects of physical function: (1) capacity for physical activities and (2) capacity for activities of daily living. Questions were selected to derive a physical function score (PFS) based on 12 items previously described by Rikli et al. [19] who developed the scale through adaptation and extension of previously published scales along with items from the American National Health Interview Survey. The 12 items include focused questions about dressing, bathing, stair climbing, household chores (cooking, cleaning and sweeping), shopping, lifting shopping, walking and pulling large objects which each received a score from 0 to 2, where 0 indicates ‘unable to do or need some help’, 1 indicates ‘can do with difficulty’ and 2 indicates ‘can do without difficulty’. Additional information about lifting heavy loads was also scored from 0 to 2, according to participation being reported as ‘never’ score 0, ‘seldom’ or ‘sometimes’ score 1 and ‘often’ or ‘always’ score 2. Participating in strenuous activities such as playing sport or going to the gym was scored as either 0 or 2. All functional scores were summed to give a total PFS with a maximum of 24 and a min of 0.

Information on smoking, level of education, history of cardiovascular disease (CVD) and medication use were obtained by questionnaire. Diabetes mellitus was defined as self-reported physician diagnosis or reported use of glucose-lowering medication or an elevated measurement of HbA1c above the guideline cut-off value for diagnosis of type 2 diabetes (T2DM) (≥48 mmol/mol [>6.5%]).

### Blood and urine samples

Non-fasting blood samples were obtained in the morning of the clinic visit following an earlier light breakfast. HbA1c was measured in stored blood samples using an immunoassay (Cobas HbA1c test). Serum creatinine was measured in fresh blood samples, and glomerular filtration rate was estimated (eGFR) using these values and ethnicity and sex specific equations previously described [20].

Early morning urine samples were collected in designated sample pots sent via post and returned by the participant on the morning of their clinic visit. Albumin creatinine ratio (ACR) was calculated from albumin and creatinine measured in urine samples. A binary variable was derived describing normal or elevated (≥3 mg/mmol) ACR.

### Grip strength

Grip strength was measured from the dominant hand using a baseline hand-held pneumatic bulb dynamometer (3B Scientific, Hamburg, Germany). Three measurements were taken, and the highest achieved was considered to be the maximum grip strength.

### Aerobic capacity

Submaximal exercise capacity was measured using a 6-minute stepper test (6MST), which has previously been validated in this age group against walking pace and sub-maximal oxygen consumption achieved in the 6-minute walk test [21]. A portable expired gas analysis system including a Polar heart rate monitor (K4B2; COSMED, Rome, Italy) was used to measure breath-by-breath V̇O_2_ and heart rate during the 6MST; the highest V̇O_2_ and heart rate achieved were determined. Maximum V̇O_2_ was predicted by extrapolating the highest measured V̇O_2_ and heart rate to the age-predicted maximum heart rate (predicted max HR=220−age). The oxygen uptake efficiency slope (OUES), an index of maximum exercise performance and cardiopulmonary reserve based on submaximal exercise, was calculated as the slope of V̇O_2_ (ml/min) versus the log of ventilation (log_*e*_VE; ml/min) across all data points measured during exercise [22].

### Echocardiography

Cardiac function was measured by transthoracic echocardiographic imaging using and ultrasound machine (EPIQ 7, Phillips) fitted with a matrix array transducer (X5-1 xMATRIX, Philips) and conducted according to the American Society of Echocardiography’s (ASE) guidelines [23]. Data analysis was performed off-line (QLAB version3.3.2, Philips). The following parameters were measured: left ventricular (LV) dimensions and wall thicknesses, stroke volume, LV mass, myocardial contraction fraction (MCF), LV ejection fraction (EF), cardiac output (CO), cardiac index (CI), total peripheral resistance (TPR), peak transmitral early diastolic velocity (*E*) an average of lateral and medial peak LV longitudinal systolic (*s*′) and early relaxation (*e*′) velocities and an index of LV filling pressure (*E*/*e*′). Full methods for each parameter measured by echocardiography are provided in supplemental information (Online resource 1; Appendix 1).

### Pulse wave velocity (PWV) and intima-media thickness (IMT)

Carotid to femoral pulse wave velocity (PWV), a measure of arterial stiffness, was measured using a commercially available cuff-based device (Vicorder, Skidmore Medical, UK). Mean intima-media thickness was measured using carotid artery ultrasound imaging (Vivid I, GE, Boston) equipped with a linear-array transducer (12L-RS,GE, Boston). Further methodological detail is provided in supplemental information (Online resource 1; Appendix 1).

### Cerebral imaging

Cerebral magnetic resonance imaging (MRI) was performed on a 3T MRI scanner (Achieva, Philips Healthcare) using an 8-channel phased-array head coil. Total brain white matter hyperintensity volume (WMH) was quantified using an automated segmentation method known as Bayesian model selection [24].

### Near-infrared spectroscopy

Skeletal muscle microvascular function (change in tissue saturation index (∆TSI%) during exercise) and oxidative capacity (the time constant (*τ*)) were assessed in the lateral head of the left gastrocnemius using near-infrared spectroscopy (NIRS; Portamon, Artinis Medical Systems, the Netherlands). NIRS measurements were collected throughout the self-paced stepping exercise test and the 3-minute recovery period. Transient arterial occlusions, by inflation of a cuff proximal to the measurement site, were performed immediately post-exercise to measure oxygen consumption rates which were used to determine *τ*. *τ* is not a direct measure of skeletal muscle oxidative capacity, but it has shown good reproducibility and agreement with the established 31P-MRS method of measuring oxidative capacity [25, 26]. Further methodological detail is provided in supplemental information (Online resource 1; Appendix 1).

### Lung function

Lung function was assessed using a PC-based spirometer (microQuark, COSMED, Italy) with software (Omnia, COSMED, Italy). Forced vital capacity (FVC) was measured, and the ratio of forced expiratory volume in 1 second (FEV1) to FVC was calculated. The best of 3 attempts was accepted as the final measurement.

### Statistical analysis

Statistical analysis was carried out in STATA 15 (StataCorp College Station, TX, USA). Categorical data are presented as *n* (%). Continuous data were examined for normality; normally distributed sample data are summarized as means ± SD and skewed data as medians (interquartile range). Comparison of sexes was done using an unpaired Student’s *t* test or a Mann–Whitney *U* test as appropriate for continuous data and *χ*^2^ test for categorical data.

Multivariable linear regression was used to adjust associations for potential confounding factors; if necessary, data were transformed to satisfy the assumptions of linear regression. Data from multivariable linear regression models are presented as beta coefficients (95% confidence intervals). The basic model (model 1) controlled for age and sex. A sex interaction term was included in model 1 to examine whether associations differed by sex. Ethinicity was adjusted for in model 2, based on prior evidence for an ethnic difference in capacity for ADLs. Further adjustment for the following potential confounders was also performed: habitual smoking behaviour and education level (model 3), type 2 diabetes (T2DM) and cardiovascular disease (CVD) (model 4).

Relative effects were examined using standardized models including one representative factor from each physiological system; cardiovascular (separated into a factor from each of cardiac systolic and diastolic function and macrovascular and microvascular function), muscle function and pulmonary function . Where multiple factors were measured, the factor with the highest standardized β coefficient for its individual association with PFS was included. Standardized models were also adjusted for all confounders described in models 2–4 (above) and in order to account for body size, height and weight. Models were fitted using full information maximum likelihood (mlmv option in Stata) to impute missing variables on the assumption of missing at random. Models including aerobic capacity were limited to participants who undertook the exercise test. Standardized models were routinely stratified by sex.

## Results

### Participants

In total, 980 participants attended the SABRE study clinic. A total of 726 participants completed all questionnaires necessary to calculate a PFS and reported their ethnicity as either European, South Asian or African Caribbean (mean age, 73±7 years; male, 57%). Participant characteristics are given in Table 1 stratified by sex. A sub-group of participants additionally undertook an exercise test (*n*=531); expired gases were measured during exercise (*n*=474) permitting estimation of maximum aerobic capacity. Participant characteristics for the sub-group are provided in full in the supplemental information file (Online resource 1; Appendix 2).

**Table 1:** Participant characteristics stratified by sex. Values are means ± standard deviation unless otherwise indicated. * indicates a skewed distribution (median(IQR)). Where data are missing from the full sample the number of samples is given in parenthesis. *ACR* albumin-creatinine ratio, *CI* cardiac index, *CVD* cardiovascular disease, *EF* ejection fraction, *eGFR* estimated glomerular filtration rate, *FEV1* forced expired volume in 1 minute, *FVC* forced vital capacity, *IMT* intima-media thickness, *MCF* myocardial contraction fraction, *PFS* physical function score, *PWV* pulse wave velocity, *TPR* total peripheral resistance, *TSI* tissue saturation index, *OUES* oxygen uptake efficiency slope, *V̇O*_*2*_ whole-body oxygen consumption

Participant characteristics	Mean ± SD, median (IQR) or no (%)				
	*n*	Women	*n*	Men	*p*
Age (years)	316	71±7	410	75±6	<0.001
Weight (kg)	316	72.7±14.1	410	79.7±13.4	<0.001
Height (cm)	316	158.4±6.3	410	170.8±6.8	<0.001
BMI (kg/m^2^)	316	28.5±5.1	410	27.3±4.1	<0.001
WHR	316	0.90±0.07	408	1.0±0.07	<0.001
Ethnicity (E/SA/AFC)	316	134/86/96 (42/27/30)	410	229/131/50 (56/32/12)	<0.001
Years of education	290	12.5±3.4	384	12.5±3.9	0.785
Current smoker	315	9 (3%)	404	14 (4%)	0.646
Diabetes	316	73 (23%)	410	95 (23%)	0.982
CVD	309	20 (6%)	404	78 (19%)	<0.001
PFS*	316	19 (18, 21)	410	21 (19, 22)	<0.001
MCF	229	43±11	294	42±10	0.048
EF (%)	229	68±9	294	65±10	0.004
*s*′ (m/s)	294	7.3±1.1	384	7.9±1.6	<0.001
CI (L/m^2^)	228	2.1±0.53	294	2.2±0.53	0.358
*E* (cm/s)	283	69±15	378	68±18	0.352
*E/e*′*	274	9.1 (7.7, 11.1)	366	8.4 (7.2, 10.1)	<0.001
PWV (m/s)	291	10.8±2.3	377	11.5±3.3	<0.001
TPR (mmHg/L)	228	29.5±8.9	294	26.4±8.6	<0.001
IMT (mm)	302	0.85±0.21	398	0.92±0.21	<0.001
∆TSI% exercise	180	−1.7±4.1	261	−3.2±4.6	<0.001
ACR elevated (≥3), *n* (%)	308	26 (8%)	403	52 (13%)	0.059
eGFR (ml/min/1.73 m^2^)	304	81.6±16.1	399	75.1±14.8	<0.001
WMH vol. (ml)*	229	2.43 (1.38, 5.78)	328	3.14 (1.50, 8.95)	0.017
FVC (L)	254	2.45±0.65	347	3.41±0.84	<0.001
FEV1 (L)	256	1.88±0.50	353	2.52±0.67	<0.001
FEV1:FVC ratio	254	0.78±0.11	347	0.75±0.09	<0.001
Est. V̇O_2_ max (ml/min/kg)	198	21.8±3.2	276	22.2±2.9	0.133
OUES (ml/min/(logL/min))	203	1.40±0.34	271	1.79±0.42	<0.001
Grip strength (kPa/kg-weight)	312	0.81±0.23	405	1.02±0.28	<0.001
Muscle oxidative capacity (s)*	37	45 (32, 68)	107	43 (29, 61)	0.355

### Physical function score

Individual items on the physical function score were compared by sex. In general, women were more likely to report inability to ‘walk 1 mile’ and difficulty or inability with ‘pulling large objects’ compared with men. A breakdown of the distribution of scores for each item, stratified by sex, is provided in the supplemental information file (Online resource 1; Appendix 3).

### Cardiovascular factors individually associated with PFS

After adjustment for age and sex, the following cardiovascular factors were associated with PFS (Table 2, model 1): cardiac function (cardiac index (CI), *E*/*e*), renal microvascular function (ACR, eGFR), cerebral microvascular health (WMH volume) and aerobic capacity (V̇O_2_max, OUES). Evidence of sex interaction was identified for systolic function (*s*′) (p=0.026), PWV (*p*=0.087) and ACR (*p*<0.001) (Table 2, column 3). Adjusting individual models for ethnicity, smoking behaviour and education level had very little effect on coefficients (Table 2, model 3). Further adjustment for confounders CVD and T2DM attenuated associations between *E*/e′ and eGFR and PFS but had little effect on the strength of association between cardiac output, ACR, WMH volume, aerobic capacity and PFS (Table 2, model 4).

**Table 2:** Individual associations between PFS and measures of cardiovascular and muscle function. *ACR* albumin-creatinine ratio, *CI* cardiac index, *eGFR* estimated glomerular filtration rate, *EF* ejection fraction, *FEV1* forced expired volume in 1 minute, *FVC* forced vital capacity, *IMT* intima-media thickness, *MCF* myocardial contraction fraction, *PFS* physical function score, *PWV* pulse wave velocity, *TPR* total peripheral resistance, *TSI* tissue saturation index, *OUES* oxygen uptake efficiency slope, *V̇O*_*2*_ whole-body oxygen consumption, *WMH* white matter hyperintensities

Association with composite physical function score	β (95% CI)	*p*	*p*	β (95% CI)	*p*	β (95%CI)	*p*	β (95% CI)	*p*	Standardized β (95% CI)
	M1: age/sex		Sex int.	M2: M 1+ ethnicity		M3: M2 + current smoker + education		M4: M3 + T2DM + CVD		(M4 adjusted)
Cardiac function										
MCF (*n*=523)	−0.007 (−0.033, 0.019)	0.612	0.512	−0.010 (−0.036, 0.016)	0.471	−0.010 (−0.036, 0.017)	0.471	−0.011 (−0.036, 0.015)	0.425	−0.034 (−0.119, 0.050)
EF (%) (*n*=523)	0.009 (−0.019, 0.037)	0.512	0.134	0.014 (−0.014, 0.042)	0.335	0.014 (−0.015, 0.042)	0.350	0.014 (−0.015, 0.042)	0.348	0.041 (−0.04, 0.13)
CI (L/m^2^) (*n*=522)	−0.68 (−1.17, −0.19)	0.007	0.431	−0.68 (−1.17, −0.19)	0.006	−0.69 (−1.18, −0.20)	0.006	−0.71 (−1.19, −0.21)	0.005	−0.12 (−0.20, −0.04)
*s*′ (cm/s) (*n*=678)	0.16 (−0.01, 0.33)	0.067	0.026	0.12 (−0.05, 0.30)	0.169	0.12 (−0.05, 0.30)	0.167	0.097 (−0.076, 0.270)	0.273	0.043 (−0.034, 0.120)
*e*′ (cm/s) (*n*=659)	0.13 (−0.02, 0.28)	0.097	0.623	0.12 (−0.04, 0.27)	0.142	0.12 (−0.04, 0.27)	0.139	0.087 (−0.064, 0.239)	0.259	0.045 (−0.03, 0.122)
LAvol. Indexed BSA (ml/m^2^) (*n*=593)	−0.033 (−0.082, 0.016)	0.189	0.716	−0.033 (−0.082, 0.016)	0.186	−0.034 (−0.082, 0.016)	0.189	−0.010 (−0.059, 0.039)	0.692	−0.016 (−0.097, 0.064)
E/e’ (*n*=640)	−0.109 (−0.203, −0.015)	0.024	0.714	−0.10 (−0.20, −0.006)	0.036	−0.10 (−0.20, −0.007)	0.036	−0.075 (−0.17, 0.019)	0.120	−0.061 (−0.138, 0.016)
Vascular function										
PWV (m/s) (*n*=668)	−0.35 (−0.12, 0.05)	0.441	0.087	−0.030 (−0.117, 0.058)	0.506	−0.030 (−0.117, 0.058)	0.513	−0.034 (−0.120, 0.051)	0.431	−0.030 (−0.105, 0.045)
TPR(mmHg/L) (*n*=522)	0.024 (−0.006, 0.054)	0.122	0.824	0.024 (−0.006, 0.055)	0.114	0.025 (−0.006, 0.055)	0.113	0.024 (−0.006, 0.055)	0.116	0.068 (−0.017, 0.153)
IMT (mm) (*n*=700)	0.58 (−0.64, 1.80)	0.351	0.814	0.65 (−0.56, 1.87)	0.293	0.65 (−0.57, 1.87)	0.296	1.05 (−0.15, 2.25)	0.088	0.065 (−0.010, 0.14)
∆TSI% exercise (*n*=441)	−0.013 (−0.066, 0.040)	0.627	0.612	−0.015 (−0.068, 0.038)	0.577	−0.016 (−0.069, 0.037)	0.541	−0.022 (−0.074, 0.031)	0.418	−0.037 (−0.13, 0.05)
ACR >3 mg/mmol (*n*=711)	−2.29 (−3.06, −1.53)	<0.001	<0.001	−2.27 (−3.03, −1.51)	<0.001	−2.27 (−3.03, −1.51)	<0.001	−2.08 (−2.84, −1.32)	<0.001	−0.19 (−0.26, −0.12)
eGFR (*n*=703)	0.021 (0.004, 0.038)	0.016	0.927	0.023 (0.006, 0.040)	0.007	0.023 (0.006, 0.040)	0.008	0.016 (−0.001, 0.033)	0.066	0.075 (−0.005, 0.155)
WMH vol. (ml) (Log) (*n*=557)	−0.35 (−0.59, −0.11)	0.004	0.705	−0.34 (−0.58, −0.11)	0.004	−0.35 (−0.058, −0.11)	0.004	−0.31 (−0.54, −0.08)	0.009	−0.11 (−0.20, −0.03)
Lung function										
FVC (*n*=601)	0.68 (0.35, 1.00)	<0.001	0.556	0.71 (0.36, 1.07)	<0.001	0.71 (0.36, 1.07)	<0.001	0.62 (0.27, 0.97)	0.001	0.18 (0.08, 0.28)
FEV1 (*n*=609)	0.74 (0.33, 1.15)	<0.001	0.223	0.72 (0.30, 1.15)	0.001	0.73 (0.30, 1.16)	0.001	0.64 (0.21, 1.07)	0.003	0.14 (0.05, 0.24)
FEV1:FVC ratio (*n*=601)	−0.77 (−3.20, 1.67)	0.536	0.326	−0.53 (−3.00, 1.94)	0.673	−0.53 (−3.00, 1.94)	0.674	−0.14 (−2.57, 2.29)	0.911	−0.005 (−0.08, 0.08)
Muscle function										
Grip (kPa) (*n*=717)	0.043 (0.030, 0.056)	<0.001	<0.001	0.045 (0.031, 0.058)	<0.001	0.045 (0.032, 0.058)	<0.001	0.041 (0.028, 0.054)	<0.001	0.26 (0.18, 0.34)
Grip (kPa/kg−weight) (*n*=717)	3.44 (2.52, 4.37)	<0.001	<0.001	3.57 (2.64, 4.50)	<0.001	3.58 (2.65, 4.50)	<0.001	3.18 (2.25, 4.11)	<0.001	0.26 (0.19, 0.33)
Grip (kPa/kg−lean mass) (*n*=651)	1.78 (1.16, 2.40)	<0.001	0.087	1.87 (1.26, 2.49)	<0.001	1.88 (1.27, 2.50)	<0.001	1.68 (1.07, 2.29)	<0.001	0.20 (0.13, 0.28)
Oxidative capacity (tau) (*n*=144)	−0.005 (−0.019, 0.009)	0.464	0.019	−0.004 (−0.018, 0.010)	0.573	−0.004 (−0.018, 0.009)	0.538	−0.002 (−0.015, 0.011)	0.801	−0.02 (−0.18, 0.14)
Aerobic capacity										
Est. V̇O_2_max (ml/min) (*n*=474) (x10^−4^)	6.8 (−0.8, 14.0)	0.078	0.792	6.7 (−0.9, 1.42)	0.083	7.0 (−0.5, 14.7)	*0.068*	7.0 (−0.3, 15.0)	0.059	0.09 (−0.003, 0.19)
Est. V̇O_2_max (ml/min/kg) (*n*=474)	0.18 (0.10, 0.25)	<0.001	0.349	0.17 (0.10, 0.25)	<0.001	0.17 (0.10, 0.25)	*<0.001*	0.16 (0.09, 0.24)	<0.001	0.19 (0.10, 0.28)
OUES (ml/min/(logL/min)) (*n*=474)	1.46 (0.87, 2.05)	<0.001	0.481	1.42 (0.83, 2.01)	<0.001	1.42 (0.83, 2.02)	*<0.001*	1.40 (0.82, 1.98)	<0.001	0.23 (0.14, 0.32)

### Pulmonary and skeletal muscle factors individually associated with physical function score

After adjustment for age and sex, FVC and grip strength were associated with PFS (Table 2, model 1). Evidence of sex interaction was identified for grip strength (*p*<0.001) and skeletal muscle oxidative capacity (*p*=0.019) (Table 2, column 3). Further adjustment for confounders had little effect on the strength of association between peripheral factors and PFS (Table 2, model 4).

### Relative contributions to PFS

**Fig. 1:**
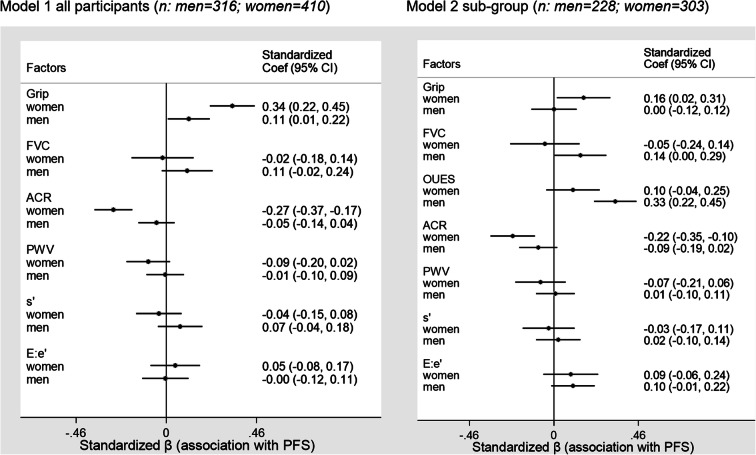
Forest plots show standardized beta coefficients representing the relative association between each factor and physical function score (PFS) in sex-stratified models. The left plot includes results from all participants, and vascular function is divided into microvascular and macrovascular representative measures (albumin-creatinine ratio (ACR) and pulse wave velocity (PWV), respectively), and cardiac function is divided into systolic and diastolic representative measures (s′ and E:e′, respectively). The right plot includes results only from the sub-group of participants who completed an exercise test permitting aerobic capacity (oxygen uptake efficiency slope (OUES)) to be included in this model. In addition to the exposures shown each model was also adjusted for: age, ethnicity, height, weight, education, presence of cardiovascular disease, presence of T2DM and smoking status. FVC forced vital capacity

Standardized β coefficients calculated for individual associations (Table 2, final column) were used to construct two standardized models that examined the relative contribution of each system to physical function stratified by sex. Vascular function was accounted for using separate measures to represent microvascular and macrovascular functions, and cardiac function was accounted for with separate measures of systolic and diastolic function (*s*′ and *E*:*e*′, respectively). In standardized model 1, after adjustment for confounders, grip strength showed an independent positive association with PFS, particularly in women where the coefficient was three times greater than in men (Fig. 1, left). Microvascular dysfunction (elevated ACR) had a strong negative association with PFS in women but not in men; the coefficient was over 5 times greater in women versus men (Fig. 1, left). There was no strong evidence that macrovascular dysfunction (elevated PWV representing increased arterial stiffness) or cardiac diastolic function was associated with PFS in these models. Cardiac systolic function (*s*′) and lung function (FVC) were positively associated with PFS in men but negatively associated with PFS in women; however, the 95% confidence intervals include the null for both estimates in this fully adjusted model (Fig. 1, left).

Standardized model 2 includes additional adjustment for a measure of aerobic capacity (OUES) and includes only participants who undertook the exercise test (*n*=531). In this sub-group, aerobic capacity was strongly associated with PFS in men (over 3 times stronger than the association in women) (Fig. 1, right). The association between grip strength and PFS, seen in model 1, was not observed for men in model 2, and the coefficient was attenuated in women. There was some evidence for a positive association between cardiac diastolic function (*E*:*e*′) and PFS in men. As in model 1, elevated ACR was strongly associated with PFS in women. The pattern of estimates for the associations between FVC, PWV and systolic function and PFS was not different from model 1 (Fig. 1, right).

As a sensitivity analysis, to examine whether the association between grip strength and PFS was present in the sub-group of participants who undertook exercise, model 1 was also performed in the sub-group without adjustment for an aerobic capacity measure. In this analysis, grip strength and ACR remained independently associated with PFS in women (standardized β coefficient (95% CI) 0.17 (0.02, 0.31), *p*=0.025, and −0.24 (−0.36, −0.12), *p*<0.001, respectively) but not in men (standardized β coefficient(95%CI) −0.02 (−0.15, 0.11), *p*=0.811, and −0.09 (−0.20, 0.02), *p*=0.100, respectively). The forest plot for the sensitivity model is provided in the supplemental information file (Online resource 1; Appendix 4).

## Discussion

### Key findings

Cardiovascular, skeletal muscle and pulmonary factors each contribute to self-reported physical function in older adults, but the relative pattern of contribution differs by sex. Grip strength is three times more strongly associated with physical function in women compared with men, while cardiorespiratory fitness is three times more strongly associated with physical function in men versus women. Microvascular dysfunction, as measured by elevated ACR, has a strong negative effect of physical function in women but this was not observed for men.

### Skeletal muscle contribution to PFS

The strong association between grip strength and physical function persisted after adjustment for confounding factors and was observed across all participants in both weight and lean mass adjusted measurements. This finding is in line with previous studies [10, 11]. In addition, our study provides novel evidence that sex modifies this association and that, in women, relative to other factors, grip strength has the strongest association with physical function suggesting preservation of muscle strength in women is a particularly important determinant of physical function in older age. The simplest explanation for this difference in association is that men have greater reserve in strength which protects them from the decline with age. Furthermore, prior observations suggest sex differences in the pattern of myofibre atrophy with age; in women, age-related decline in muscle mass is largely due to type II (fast-twitch) myofibre atrophy, whereas, in men, although the number of myofibres declines, type II fibres are generally not atrophic, and type I fibres undergo hypertrophy to compensate [27]. Although we adjusted for differences in body habitus between men and women, we did not measure myofibre types, so this explanation remains speculative. Other factors related to muscle quality, such as fat infiltration and fibrosis, and muscle function (neuro-muscular drive) may also influence the association between grip strength and physical function differentially by sex. These factors merit further investigation in future studies. In contrast to previous studies [4, 12], we did not detect an association between skeletal muscle oxidative capacity and physical function. This could be explained by the different outcome measures used, walking speed versus our composite physical function score, or by the methodological differences in measures of oxidative capacity. While our study included a non-invasive optical method (NIRS) to measure of oxidative capacity, others used ^31^P magnetic resonance spectroscopy (MRS) to directly capture PCr recovery or muscle biopsies to examine specific components of muscle energetics individually, such as the activity of specific enzymes.

### Cardiovascular contribution to PFS

We observed a positive association between cardiorespiratory fitness (OUES) and physical function, in line with previous studies [5, 28]. We examined OUES in addition to V̇O_2_max, as OUES is considered more representative of overall cardiac and vascular function [22]. The finding that, relative to grip strength, OUES is more strongly associated with physical function in men is novel. Paterson et al. previously described similar relative associations, but they did not present sex-stratified results [28]. One potential explanation for this finding is that cardiorespiratory fitness mediates some of the association between grip strength and physical function. However, as our sub-group only included participants free from contra-indicators for exercise, these participants were generally in better health than the overall sample. In the sensitivity analysis, conducted in the sub-group of participants who undertook exercise without adjustment for aerobic capacity, there was also no evidence of an association between grip and PFS in men and the coefficient for women was half of that in the model for the whole sample, suggesting an alternative explanation that there is heightened reserve in grip strength in more healthy sub-groups of the population.

Previously, higher inflammation (elevated circulating levels of CRP, IL-6 and IL-1RA) has been associated with lower levels of physical function [29]. Although it is difficult to indicate a single mechanism, there is potential for any of the systems investigated here to mediate this association. Furthermore, it is possible that the effects of inflammation on physiological systems differs by sex and could thus explain some of the sex differences we have observed. Another likely scenario is that inflammation exaggerates changes in body composition typical of aging which negatively impacts physical function. While we adjusted for body size in general (height, weight and lean mass), a more detailed investigation into the sex differences in muscle and adipose distribution may help to explain the sex differences observed here. Men were older than women in this study, and, although we accounted for chronological age, this may not fully account for potential sex differences in the age-related rates of inflammatory processes.

An elevated urinary ACR (albuminuria; >3m g/mmol) is widely regarded as a marker of glomerular microvascular dysfunction that predicts incident CVD. In this study, elevated ACR was negatively associated with physical function. Similarly, elevated ACR doubles the risk of disability [30] and is related to increased frailty [31]. We also observed that this association was robust to adjustment for CVD and measures of cardiac and macrovascular functions suggesting that independent microvascular mechanisms may be involved. Elevated ACR has also been linked to cerebral microvascular dysfunction [32] negatively affecting motor function (potentially via cortical damage or atrophy). Therefore, an alternative explanation is impaired motor control negatively affects physical function. We also showed an individual inverse association between cerebral WMH volume (indicating microvascular damage) and PFS, and we did not find an association between ∆TSI% (indicating the capacity for skeletal muscle microvascular perfusion during exercise) and physical function.

We present further novel evidence that the association between microvascular dysfunction and physical function is 5 times greater in women versus men. This suggests that the pre-clinical manifestations of microvascular dysfunction may affect women at a lower threshold than men which is in line with the differential pathophysiology of heart failure in women [15]. However, we did not detect sex interactions for the cerebral or skeletal muscle microvascular measures with physical function. Further work is necessary to better understand the mechanistic underpinning of the difference in microvascular dysfunction-physical function association between men and women.

We were unable to find a strong association between macrovascular measures (IMT and vascular stiffness (PWV)) and physical function in men or women after adjustment for confounders and other central and peripheral factors. This supports some previous work [33], and findings showing no association between coronary artery calcification score (CACS) and physical function [34]. Conversely, others have shown an inverse association between CACS and walking speed in men (but not in women) [35]. Our results indicate a weak positive association between cardiac systolic function and PFS in men which could be the result of systolic dysfunction secondary to ischemic damage.

This analysis was performed using cross-sectional data; therefore, we are unable to determine the direction of these associations. On the one hand, poor physical function reflects a lack of participation in physical activity, a well-established risk factor for CVD [36]. Therefore, the associations observed between cardiovascular factors and PFS could reflect the cardio-protective effect of higher physical activity levels. Alternatively, clinical and sub-clinical cardiac and vascular impairments may lead to impaired physical function via mechanisms such as a reduced capacity to deliver oxygenated blood to skeletal muscle, a negative effect on cerebral blood flow and thus damped motor function or via indirect effects (such as negative consequences of sustained inflammation). Nevertheless, these findings provide novel insight into factors most strongly related to physical function in a sample reasonably representative of older adults living in the UK. A firm understanding of the determinants of poor physical function in older adults is relevant in the design of targeted novel approaches that sustain independent living in later life, thus reducing disability and the burden of social care. The sex differences described here suggest that sex-specific interventions may be appropriate in addressing loss of physical function with age.

## Conclusions

Cardiovascular, skeletal muscle and pulmonary factors each contribute to physical function in older adults, but this study provides evidence that the relative pattern of contribution differs in men versus women. This has important implications for design of exercise training programs to mitigate the decline in physical function suggesting that sex-specific targets may increase effectiveness.

## Supplementary information

ESM 1 (PDF 706 kb)
