# Supplementary material for: Sex differences in the contribution of different physiological systems to physical function in older adults
Source: GeroScience. 2021 Feb 11;43(1):443–55. doi: 10.1007/s11357-021-00328-y (PMC8050191; doi:10.1007/s11357-021-00328-y)
Supplement: Supplementary file 1 — (PDF 706 kb) [file 11357_2021_328_MOESM1_ESM.pdf]

Supplemental information

Online resource for the following manuscript:

Title: Sex differences in the contribution of different physiological systems to physical function in older adults

Authors: Siana Jones, Martin G Schultz, Therese Tillin, Chloe Park, Suzanne Williams, Nishi Chaturvedi & Alun D Hughes

Corresponding author: Siana Jones, MRC Unit for Lifelong Health & Ageing at UCL, Department of Population Science & Experimental Medicine, Institute for Cardiovascular Science, University College London

Corresponding author contact: siana.jones@ucl.ac.uk

Appendix 1

Echocardiography extended methods

Cardiac function was measured by transthoracic echocardiographic imaging (EPIQ 7, Phillips) fitted with a matrix array transducer (X5-1 xMATRIX, Philips) and conducted according to the American Society of Echocardiography's (ASE) guidelines.(1) The examination was performed by one of two experienced sonographers. Participants were in the left lateral decubitus position, ECG leads were placed to ensure a clear QRS complex throughout image acquisition. Data analysis was performed off-line (QLAB 3.3.2, Philips). Further details of the methods for echocardiography are provided in previously published work. (2, 3)

Left ventricular (LV) dimensions and wall thickness were measured from the parasternal long axis window. LV end systolic (ESV) and diastolic (EDV) volumes were calculated using the Teichholz formula (4) and stroke volume was calculated as EDV minus ESV. LV mass was estimated according to ASE recommendations.(1) Myocardial volume was estimated from the LV mass estimation (see above) divided by the density of myocardial tissue (1.04 g/ml). Myocardial contraction fraction (MCF) was calculated as the ratio of stroke volume to myocardial volume.(5) Ejection fraction (EF) and cardiac output (CO) were estimated from ESV, EDV and resting heart rate (CO only). Cardiac index (CI) was calculated as CO/body surface area (BSA). Total peripheral resistance (TPR) was estimated from a clinical brachial blood pressure measurement of mean arterial pressure (MAP) and CO measured by echocardiography.

The mitral inflow pattern was imaged using spectral pulsed wave Doppler with sample volume placed at the tips of the mitral valve inside the LV. Peak early diastolic velocity (E) was measured from traces. Wall motion was measured using Tissue Doppler imaging (TDI) at the level of the mitral valve in the 4 chamber window, an average of lateral and medial peak longitudinal systolic (s') and early relaxation (e') velocities were calculated. An index of LV filling pressure was calculated as E/e'.

Pulse wave velocity (PWV) & intima-media thickness (IMT) extended methods

Carotid to femoral Pulse Wave Velocity (PWV), a measure of arterial stiffness, was measured using a commercially available cuff-based device (Vicorder, Skidmore Medical, UK). Intima-Media thickness was measured using carotid artery ultrasound imaging (Vivid I, GE, Boston) equipped with a linear-array transducer (12L-RS,GE, Boston). Settings were adjusted to obtain optimum image quality ensuring that the carotid bifurcation and the intima and media at both the near and far wall were visible over a length of at least 10 mm in the common carotid artery. A cine loop of at least 5 cycles at 3 angles (lateral, posterior and anterior) on each side was captured. Measurements were made offline

using validated software (AMS II). The region of interest was defined as 1 cm proximal to the bifurcation.

Near-infrared Spectroscopy extended methods

Skeletal muscle microvascular function and oxidative capacity were assessed using near infrared spectroscopy (NIRS) measurements of changes in oxygenated and deoxygenated haemoglobin and tissue saturation index (TSI%) during exercise and arterial occlusions. The NIRS device (Portamon, Artinis Medical Systems, the Netherlands) was positioned on the lateral head of the gastrocnemius where the calf girth was greatest. The difference between TSI% at rest and at the end of exercise was calculated. Greater absolute reductions in this value for a given workload during exercise are thought to represent haemodynamic insufficiency.(6) Immediately post-exercise, a rapid inflation cuff (Hokanson, SC10D/E20; PMS Instruments, Maidenhead, UK) placed on the thigh proximal to the location of NIRS measurement was inflated transiently to induce complete arterial occlusions (5–8 s intervals) throughout a 3 min recovery period to determine the kinetics of muscle recovery, represented by a time constant.(7) The time constant has shown good reproducibility and agreement with the established ^{31}P-MRS method of measuring skeletal muscle oxidative capacity.(8, 9) All analysis of NIRS data was conducted off-line using custom written programs in MATLAB R2014a (MathWorks, Natick, MA, USA).

References for extended methods

1. Mitchell C, Rahko PS, Blauwet LA, Canaday B, Finstuen JA, Foster MC, et al. Guidelines for Performing a Comprehensive Transthoracic Echocardiographic Examination in Adults: Recommendations from the American Society of Echocardiography. J Am Soc Echocardiogr. 2019;32(1):1-64.
2. Park CM, Tillin T, March K, Ghosh AK, Jones S, Wright A, et al. Hyperglycemia has a greater impact on left ventricle function in South Asians than in Europeans. Diabetes Care. 2014;37(4):1124-31.
3. Park CM, March K, Ghosh AK, Jones S, Coady E, Tuson C, et al. Left-ventricular structure in the Southall And Brent REvisited (SABRE) study: explaining ethnic differences. Hypertension. 2013;61(5):1014-20.
4. Teichholz LE, Kreulen T, Herman MV, Gorlin R. Problems in echocardiographic volume determinations: echocardiographic-angiographic correlations in the presence of absence of asynergy. Am J Cardiol. 1976;37(1):7-11.
5. Maurer MS, Koh WJ, Bartz TM, Vullaganti S, Barasch E, Gardin JM, et al. Relation of the Myocardial Contraction Fraction, as Calculated from M-Mode Echocardiography, With Incident Heart Failure, Atherosclerotic Cardiovascular Disease and Mortality (Results from the Cardiovascular Health Study). Am J Cardiol. 2017;119(6):923-8.
6. Boezeman RP, Moll FL, Unlu C, de Vries JP. Systematic review of clinical applications of monitoring muscle tissue oxygenation with near-infrared spectroscopy in vascular disease. Microvasc Res. 2016;104:11-22.
7. Ryan TE, Erickson ML, Brizendine JT, Young HJ, McCully KK. Noninvasive evaluation of skeletal muscle mitochondrial capacity with near-infrared spectroscopy: correcting for blood volume changes. Journal of applied physiology. 2012;113(2):175-83.

8. Southern WM, Ryan TE, Reynolds MA, McCully K. Reproducibility of near-infrared spectroscopy measurements of oxidative function and postexercise recovery kinetics in the medial gastrocnemius muscle. Applied physiology, nutrition, and metabolism = Physiologie appliquee, nutrition et metabolisme. 2014;39(5):521-9.
9. Ryan TE, Southern WM, Reynolds MA, McCully KK. A cross-validation of near-infrared spectroscopy measurements of skeletal muscle oxidative capacity with phosphorus magnetic resonance spectroscopy. Journal of applied physiology. 2013;115(12):1757-66.

Appendix 2

Characteristic	Mean±SD, median (IQR) or n(%)				
	n	Women	*n*	Men	*p*
Age (years)	*228*	70±7	*303*	75±6	*<0.001*
Weight (kg)	*228*	71.7±13.5	*303*	79.0±12.1	*<0.001*
Height (cm)	*228*	158.8±6.4	*303*	170.8±6.9	*<0.001*
BMI (kg/m^2)	*228*	28.4±4.8	*303*	27.1±3.6	*<0.001*
WHR	*228*	0.90±0.07	*302*	1.0±0.07	*<0.001*
Ethnicity (E/SA/AFC) *n*(%)	*228*	*95/60/73* (42/26/32)	*303*	*165/99/39* (55/33/13)	*<0.001*
Years of education	*209*	12.5±3.5	*284*	12.4±3.7	*0.640*
Current smoke, *n*(%)	*227*	8(4%)	*299*	8(3%)	*0.575*
Diabetes, *n*(%)	*228*	50(22%)	*303*	67(22%)	*0.960*
CVD, *n*(%)	*222*	11(5%)	*300*	50(17%)	*<0.001*
PFS*	*228*	20(18,21)	*303*	21(19,22)	*<0.001*
MCF	*184*	44±10	*233*	42±10	*0.090*
EF (%)	*184*	68±8	*233*	66±10	*0.007*
TDI s' (m/s)	*221*	7.3±1.1	*292*	8.0±1.6	*<0.001*
CI (L/m^2)	*183*	2.1±0.50	*233*	2.2±0.53	*0.294*
E (cm/s)	*214*	69±15	*285*	67±17	*0.142*
E/e'*	*210*	9.0(7.6,11.0)	*278*	8.4(7.2,10.1)	*0.013*
PWV (m/s)	*209*	10.7±2.1	*278*	11.5±3.4	*<0.001*
TPR (mmHg/L)	*183*	28.7±8.6	*233*	25.8±7.4	*<0.001*
IMT (mm)	*226*	0.85±0.22	*302*	0.92±0.19	*0.003*
ΔTSI% exercise	*180*	-1.7±4.1	*261*	-3.2±4.6	*<0.001*
ACR elevated (≥3), n(%)	*228*	14(6%)	*303*	33(11%)	*0.056*
eGFR (ml/min/1.73m^2)	*225*	81.9±15.6	*301*	75.3±14.4	*<0.001*
WMH vol. (ml)*	*179*	2.32(1.23,5.63)	*260*	2.98(1.49,8.92)	*0.015*
FVC (L)	*191*	2.48±0.68	*260*	3.43±0.84	*<0.001*
FEV1 (L)	*193*	1.90±0.51	*265*	2.55±0.65	*<0.001*
FEV1:FVC ratio	*191*	0.77±0.12	*260*	0.75±0.09	*0.023*
Est. $\dot{V}O_2$ max (ml/min/kg)	*198*	21.8±3.2	*276*	22.2±2.9	*0.133*
OUES (ml/min/(logL/min))	*203*	1.40±0.34	*271*	1.79±0.42	*<0.001*
Grip strength (kPa/kg-weight)	*228*	0.83±0.22	*303*	1.04±0.26	*<0.001*
Muscle oxidative capacity (s)*	*37*	45(32,68)	*107*	43(29,61)	*0.355*

Appendix 3

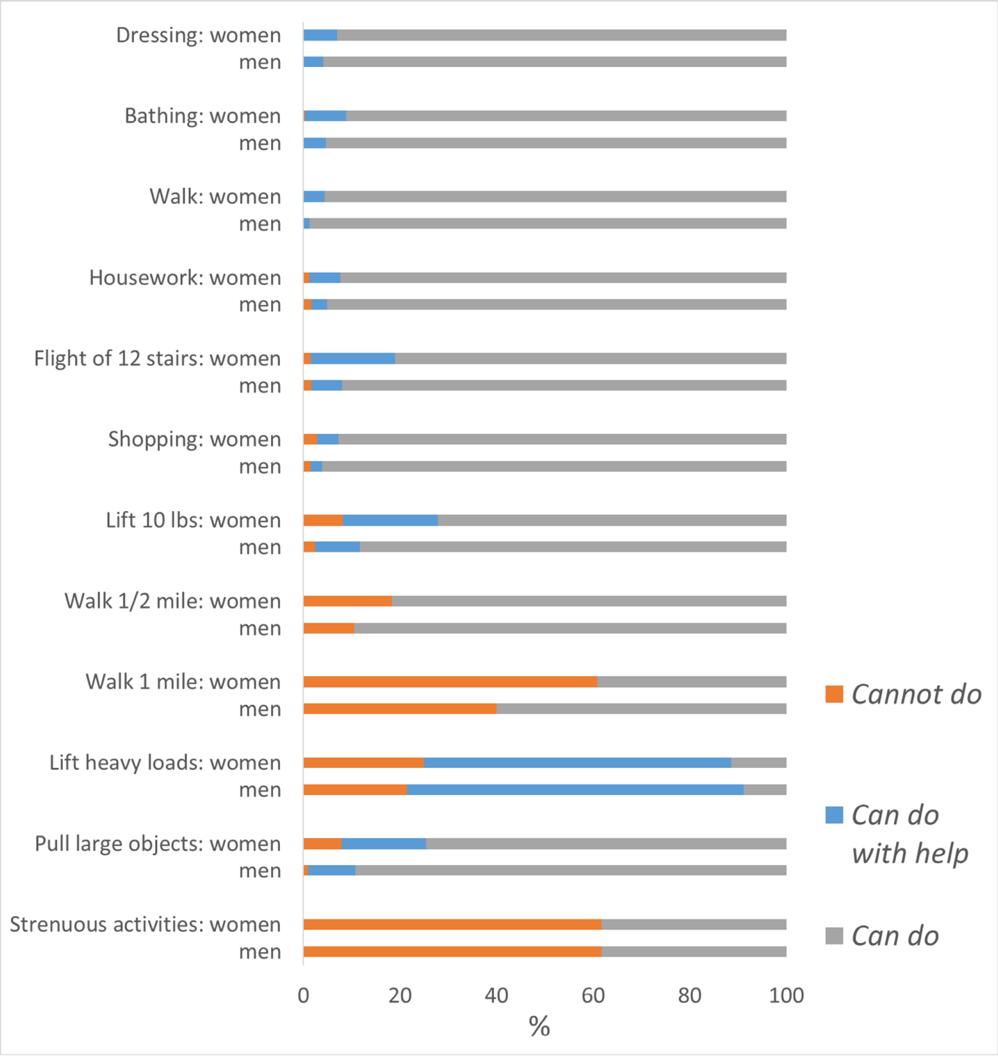

The distribution of answers to questions related to each of the 12-items necessary to calculate physical function score stratified by sex. Grey bars indicate the percentage of participants who reported they can do the activity, blue bars indicate those who can do the activity with assistance and orange bars indicate those who cannot do the activity.

Appendix 4

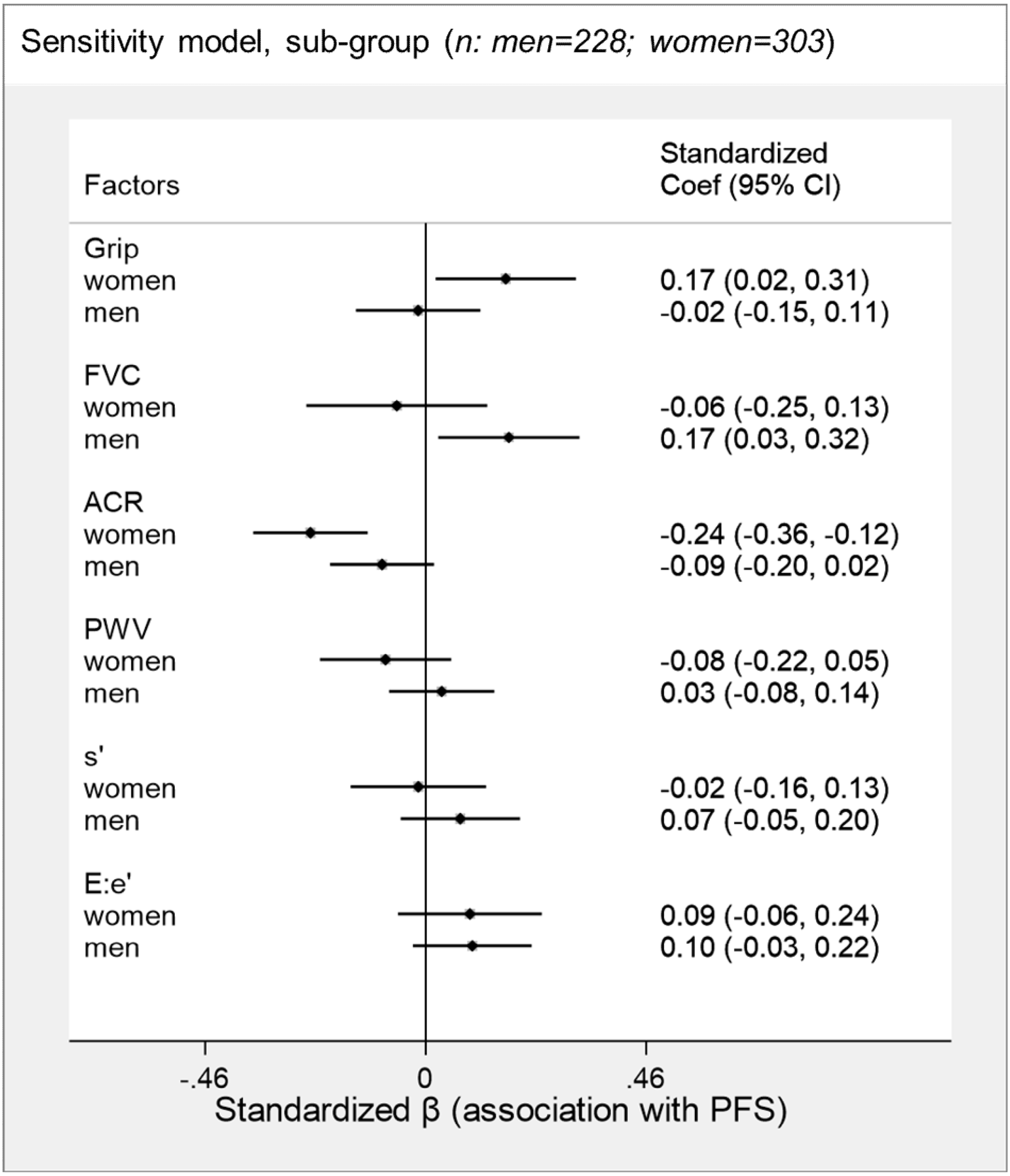

Sensitivity model, sub-group (n: men=228; women=303)
Factors
Standardized Coef (95% CI)
Grip
women
men
0.17 (0.02, 0.31)
-0.02 (-0.15, 0.11)
FVC
women
men
-0.06 (-0.25, 0.13)
0.17 (0.03, 0.32)
ACR
women
men
-0.24 (-0.36, -0.12)
-0.09 (-0.20, 0.02)
PWV
women
men
-0.08 (-0.22, 0.05)
0.03 (-0.08, 0.14)
s'
women
men
-0.02 (-0.16, 0.13)
0.07 (-0.05, 0.20)
E:e'
women
men
0.09 (-0.06, 0.24)
0.10 (-0.03, 0.22)
-.46
0
.46
Standardized β (association with PFS)
